# Detecting local establishment strategies of wild cherry (*Prunus avium *L.)

**DOI:** 10.1186/1472-6785-6-13

**Published:** 2006-10-04

**Authors:** Aki M Höltken, Hans-Rolf Gregorius

**Affiliations:** 1 Institut für Forstgenetik und Forstpflanzenzüchtung, Büsgenweg 2, 37077 Göttingen, Germany

## Abstract

**Backround:**

*P. avium*, a pioneer tree species that colonizes early forest successional stages, is assumed to require an effective strategy allowing stably repeatable rounds of local establishment, dispersal and local extinction. Consequently, the early replacement of cherry by climax tree species makes the establishment of several local generations very unlikely, especially in central European continuous cover forests. This has to be seen in connection with the mixed reproduction system involving asexual reproduction as a complementary adaptational strategy. Tests of the local establishment of wild cherry must therefore consider the possibility of first generation establishment via seedling recruitment potentially followed by an asexual generation (root suckering). Successful establishment can therefore be determined only among adult individuals with the option of detecting vegetative reproduction at these stages. To test the implied suggestion about local establishment strategies of wild cherry, nuclear microsatellites were used to analyse patterns of asexual propagation among adult stages that have been subjected to one of two major types of forest management. These management types, the historical "coppice with standards system" (CWS) and the "high forest system" (HFS), can be reasonably assumed to have affected the reproduction system of *P. avium*.

**Results:**

Clear differences were found in the reproduction pattern between two stands representing the two forest management types: 1) Clonal propagation is observed in both management systems, but with a distinctly higher frequency in the CWS. Hence, sexual recruitment as a first local generation is followed by a second asexual generation in both, whereas in the CWS there is evidence for an additional clonal generation. 2) The estimation of amounts of clonal reproduction critically depends on the assumptions about multilocus gene associations. This is revealed by the application of newly developed methods of quantifying gene associations. 3) Haplotype diversities are higher in the CWS and found to be associated with a large degree of heterozygosity for the second largest clonal group. 4) Seed set was sparse over the last eight years of observation in the CWS stand.

**Conclusion:**

This study provides useful guidelines for more comprehensive investigations, particularly on the interrelationships between degrees of cloning and capacity of sexual reproduction, amounts of multilocus gene associations, effects of heterozygosity on cloning success, and sustainability of different forest management types.

## Backround

In clonal plants, modular growth allows for the production of new individuals (ramets) that are genetically identical to each other and to their clonal ancestor [1]. This behaviour is observed in the tree species *Prunus avium *L. (wild cherry). It endows the species with a mixed sexual and asexual reproduction system in which each individual does not only produce seed but is also able to spread vegetatively by root suckers.

Typical of pioneer tree species such as wild cherry is that they colonize early forest successsional stages as a result of forest disturbances (larger gaps in the forest canopy). Although this species reaches ages of up to 80 years and more, it is generally replaced by climax tree species during progressing succession of the local disturbance (e.g. beech, *Fagus sylvatica *L.). This requires an effective strategy allowing stably repeatable rounds of local establishment, dispersal and local extinction, particularly in natural central European continuous cover forests. Consequently, the establishment of local populations including several generations is unlikely due to a lower competitive ability compared to climax tree species (e.g. limited shade tolerance, early declining growth rates). While this expectation has been confirmed by a number of observations [2], it does not explain the advantages of a mixed reproductive strategy involving clonal reproduction in particular. Moreover, central European forests are since long strongly affected by different forest management strategies, several of which do not reflect the supposed natural reproduction patterns. In fact, *P. avium *can be observed primarily in two systems of forest management, in "group selection shelterwood systems" (= high forest system, HFS in the following) and in relics of former "coppice with standards" systems (= CWS in the following).

In the group selection shelterwood systems, natural regeneration is achieved by irregular, groupwise lowering of stand density in old stands. Such gaps are gradually increased over a time span of 30 to 50 years until total clearing. This technique allows the regulation of the abundance of tree species differing in shade-tolerance and thus the mixture of species typical for various stages of forest succession. Besides single-tree selection forests, this kind of forest treatment is supposed to represent similar forest tree reproduction patterns expected for natural central European forest ecosystems without human interference.

The former CWS combined the supply of firewood as well as construction timber (two-storied system). Firewood was produced by exploiting ("= coppicing") the even aged lower storey within short intervals (5 to 40 years) promoting tree species capable of vegetative reproducetion (e.g. stump shooting, root suckering). Only some high quality trees, the so called "standards", were left (about 25 to 50 trees per hectare) leading to an uneven aged overstorey of irregularly distributed stems and irregular canopy cover [3].

Hence, in order to understand the ecological basis of the mixed sexual and asexual reproduction system of wild cherry, it is desirable to analyse local occurrences of cherry for patterns of sexual and asexual reproduction among adult individuals. The focus on the adult stage is justified by the fact that successful establishment can only be stated after individuals reached adulthood. In this way it would be possible to test the range of validity of the common hypothesis that wild cherry does not spend more than one generation at the same location.

An assessment of this hypothesis therefore critically depends on the possibility to distinguish between adult individuals derived from asexual and from sexual reproduction. Basically, suitable methods of clonal analyses are provided by genetic markers of sufficient variability, such that an efficient rejection of the hypothesis of sexual reproduction is possible. More precisely, the clonal origin of two individuals with identical multilocus-genotypes is assumed if the hypothesis is rejected that two copies of such a genotype can be produced independently by sexual reproduction. The common methods of clonal analyses are based on the absence of homologous (allelic) and non-homologous (non-allelic) gene associations [see [4-7]]. These assumptions are a priori not realized in wild cherry because of its gametophytic self-incompatibility system (GSI) in combination with its capacity for clonal propagation.

In fact, the assumption of independent association of genes is already questioned by the early work of [8] about the effects of partial self-fertilization (see also [9] for a more detailed recent treatment). Beyond this, stochastic associations among genes can be generated by all major evolutionary and adaptational processes, such as selection, mating and random drift. The present paper will therefore be concerned with the application and critical evaluation of recently developed methods that take genic associations into consideration [10].

## Results

By applying six nuclear microsatellites [11,12] (table 1) in two wild cherry stands, we obtained different proportions of distinguishable genotypes. In the stand of Roringen, which is managed as a HFS, 48 different multilocus genotypes were found among 56 analyzed adult individuals. In Wibbecke, however, a relic stand of a former CWS, we observed only 24 multilocus genotypes among 78 *P. avium *trees.

For both stands we found a mean number of 5.5 alleles per locus. The stands differed for their diversity measures: In Roringen a gene pool diversity of 2.88 (ranging from 2.01 to 4.02 among the individual loci) and in Wibbecke a gene pool diversity of 3.00 (ranging from 1.51 to 4.86 among the individual loci) was found (Table 1). Combined into the hypothetical gametic diversity, this yielded values of 649.24 and 1306.24 for Roringen and Wibbecke, respectively. This suggests generally higher efficiencies for the identification of clones in Wibbecke than in Roringen. The allele frequencies that are the basis for the following statistical calculations concerning clonal vs. sexual reproduction are listed in Table 2.

In order to obtain information whether identical multilocus genotypes belong to the same clone, the testing procedure *C*_*2*_^*N *^was applied to the common characterization of sexual reproduction by random gene association, which is determined by a relative gene association *A*_*r*_(*g*) = 0 (Table 3 and 4). The values for *C*_*2*_^*N *^ranged from 1.8033 · 10^-6 ^to 2.4674 · 10^-11 ^in Roringen and 7.9074 · 10^-6 ^to 3.1075 · 10^-13 ^in Wibbecke for all genotypes observed in at least two copies, which are consistently highly significant. Particularly low values were calculated for *C*_*n*_^*N*^, indicating that the probability of exclusively sexual reproduction within groups of identical genotypes tends towards zero. Hence, all copies of genotypes observed in both stands are suggested to be due to clonal propagation.

Based on the absence of gene associations, the degree of clonal propagation measured as the average number of individuals (ramets) per clone (genet cloning success) (i.e. *N*/*G*, with *N *= number of individuals and *G *= number of clones or genets) thus yields estimates 56/48 (= 1.166) and 78/26 (= 3.00) in Roringen and Wibbecke, respectively. These estimates are to be considered as minimum values, since, with low probability, the copies of a genotype may contain further clones.

The highly significant values for *C*_*2*_^*N*^, however, turned out to be misleading when additional markers were supplied. Such „Type one errors“ were detected for the multilocus genotypes *Ror1*, *Wib1*, *Wib3*, *Wib4 *and *Wib6 *(Table 3) after the analysis of two additional microsatellites in Roringen and six polymorphic isozymes in Wibbecke. By the above explanations this is highly likely to be due to the inappropriateness of the assumption *A*_*r*_(*g*) = 0. As shown above, removal of the error requires an increase of *A*_*r *_to at least guarantee that *C*_*2*_^*N*^(*H*) ≥ *ε*. This threshold value of *A*_*r *_was computed for each of the multilocus genotypes that occurred at least twice within the two wild cherry stands and that are thus candidates for clonal propagation.

Since all genotypes are heterozygous for at least one gene locus, the largest lower bound α equals 0 for all genotypes according to equation 1 in the additional file. The least upper bound *ω*(*g*) ranges from 0.054 to 0.268 in Roringen and from 0.038 to 0.282 in Wibbecke (see Table 4). The highest *ω*-values were obtained for multilocus genotypes for which a Type one error was detected (*ω*(*g*) ranging from 0.166 to 0.282). Among those genotypes with large *ω*-values no Type one error was detected for only three (with the exceptions *Ror4*, *Wib7 *and *Wib10*, with *ω*(*g*) ranging from 0.224 to 0.282). The majority of genotypes for which no Type one error was detected therefore showed the lowest *ω*-values. Since a small *ω*-value for a genotype indicates that its alleles are rare (and so the genotype itself), this accords with the expectation that copies of such genotypes belong to a single clone. This is also reflected by the frequencies of genotypes hypothesized to result from random gene association (see Table 4).

**Figure 1:**
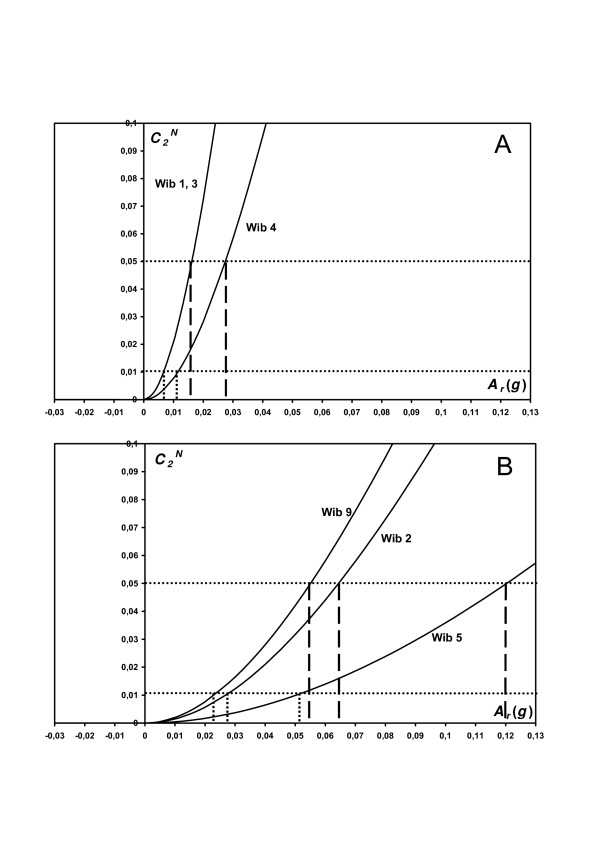
***C*_*2*_^*N *^as a function of the relative measure of gene association *A*_*r*_(*g*) computed for different multilocus-genotypes (MLG) of *P. avium *in the stand of Wibbecke**. The critical values of the relative measure of gene association *A*_*r*_(*g*) for *C*_*2*_^*N *^= *ε *(= 0.05 and 0.01) are plotted; A: MLGs of Wibbecke, for which a 'Type one error' was detected; B: Three of the 13 MLGs of Wibbecke, which were identical also for the additionally used markers (no 'Type one error' detected)

For the five genotypes for which a Type one error was detected, Table 4 also gives the lower bound of the relative gene association *A*_*r*_(*g*) that would have avoided the Type one error. These lower bounds result as the smallest value of *A*_*r *_for which *C*_*2*_^*N *^= 0.05. The bounds were in a first step obtained graphically by plotting *C*_*2*_^*N *^as a function of *A*_*r *_(see figure 1). Subsequently this value was made more precise by computational approximation. The differences in the graphs of figure 1 are due to the differences in allele frequencies and the resulting least upper bounds (*ω*(*g*)) for the respective MLGs. Because of the very small expected frequencies of the MLGs the differences in the graphs are mainly due to the differences in the least upper bounds *ω*, such that in effect the graphs become steeper with increasing *ω*.

Assuming that the largest of these lower bounds of relative gene association (*A*_*r *_= 0.0275) is not exceeded by any of the genotypes found in at least two copies, the significance probabilities *C*_*2*_^*N *^were re-calculated. The re-calculation is based on the genotypes determined for the initial six microsatellite loci. The result is shown in the rightmost column of Table 4, and it demonstrates that it cannot be ruled out that three of the genotypes for which a Type one error was not detected for *A*_*r *_= 0 consist of more than one clone at the significance level ε = 0.05. These three genotypes are exactly those which complete the group of genotypes with the distinctly largest *ω*-values.

The stand with the larger degree of clonal propagation (Wibbecke) is also the one with the higher measures of genetic diversity for both the gene pool and the hypothetical gametic pool (see Table 1). Since, as was mentioned above, higher genetic diversity increases the likelihood of detecting clones, this hints at the possibility that the number of clones detected in Roringen is underestimated. The reasoning of this expectation lies in the fact that only among genetically identical individuals additional ramets belonging to different clones can be expected to exist, and that this possibility is indicated by a significance probability that exceeds the level of significance (i.e. *C*_*2*_^*N *^≥ ε). Yet, nothing definite can be said about the actual number of clones present among the copies of a genotype for which *C*_*2*_^*N *^≥ ε holds. Hence, in the test based on *A*_*r *_= 0 this is irrelevant, since for all genotypes with at least two copies the test yields significance (i.e. *C*_*2*_^*N *^< ε). It does, however, become relevant under the assumption *A*_*r *_= 0.0275 for the inital six microsatellite loci.

Calculating cloning success in the two stands with all the additional information (application of further markers and considering gene association), we revealed slightly modified results for the HFS (*N*/*G *= 56/49 = 1.143 instead of 1.166) and larger differences for the CWS (*N*/*G *= 78/34 = 2.290 instead of 3.000). The same holds for Simpson's index of concentration *C*. The probability to draw two individuals with the same MLG decreases for both stands, slightly for the HFS (from *C *= 0.008 to *C *= 0.007) and distinctly for the CWS (from *C *= 0.067 to 0.051).

**Figure 2:**
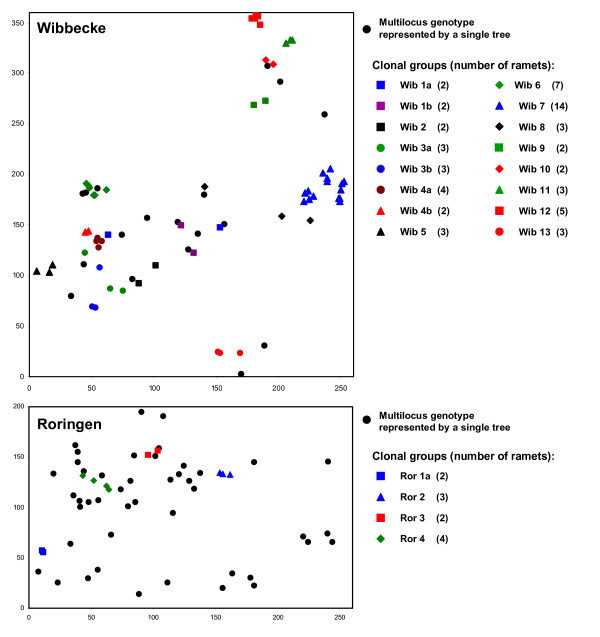
**Map of the studied *P. avium *(wild cherry) stands**. Location of adult *P. avium *(wild cherry) genotypes represented by a single tree and by groups of trees (clonal groups)

Looking at the map in figure 2, the clonal groups in Roringen are found in clusters or chains, whereas the distribution of the ramets of several single clones in Wibbecke shows larger spatial spread (about 90 m for the genet Wib 1a and about 75 m for Wib 8) and intermingling (Wib 3a, Wib 3b).

## Discussion

Woody plant species are able to produce potential embryonic meristems on different parts of the plant body. In temperate broad-leaved forests, the most effective system of clonal growth is displayed by root suckering (e.g. members of the species *Populus*, *Salix *and *Prunus*). Similarly to herbaceous plants, these meristems often behave as opportunistic organs securing colonization of unoccupied sites or increasing competitive power of the species within the community [13]. In this study we are particularly interested in the effects of two different forest management strategies on sexual vs. asexual reproduction and genetic diversity of the forest tree species *Prunus avium *L. (wild cherry). We are, however, aware of the problems with classical statistical methods of testing for clonal propagation that result from questionable assumptions on how sexual reproduction determines the association of genes in genotypes. Particularly for the known characteristics of the reproductive system of wild cherry, these assumptions seem to be unrealistic and may therefore lead to biased assessments of the role of clonal propagation in this species.

Two stands were chosen for our analysis, one about 70 years old (Roringen) that is managed as a HFS (a mixture of shelterwood and selective thinning), and a second stand (Wibbecke) that is about 100 years old and was formerly managed as CWS. The results of differrent man-made ecological conditions and, consequently, different establishment histories of the two stands are reflected in typical species compositions. Although the CWS is largely out of practice since the beginning of the last century, species such as *Quercus petraea *(sessile oak), *Carpinus betulus *(hornbeam) and *Tilia cordata *(basswood) are still dominating the forest of Wibbecke. According to [14] these species are better adapted to late (spring) frost and drought, since the density of the canopy was irregular under coppice regimes, covering only a small proportion of the area. Furthermore, these species are capable of sprouting (stump shooting) after (man-made) injury and, in the case of wild cherry, are even able to produce new individuals (ramets) by root suckers. They also contribute to forest tree species diversity.

In contrast, in the HFS, shade-tolerant species such as *Fagus sylvatica *(common beech) are the dominating species and by this reduce forest tree species diversity. Light-demanding species such as *P. avium *mainly colonize early successional stages and are thus dependent on gaps in the forest canopy. Such gaps (up to 60 m in diameter) are, for example, provided in shelterwood forests, where a group selection method of cutting is applied.

The conditions for establishment of wild cherry are thus distinctly different between the two management systems and can be expected to affect the mode of reproduction. In fact, our observations on clonal reproduction lend support to this expectation in special ways. The initially posed question as to the formation of more than one local generation can be answered differently for the two management systems. The individual cloning success as well as the degree of cloning at the stand level are distinctly higher in the CWS (*N*/*G *is doubled and *C *is even seven times larger). This indicates that in HFS, as compared to CWS, the chances are strongly reduced to reach the canopy in a second local vegetative generation. There are clear indications that this is different in the CWS, particularly in view of the considerable spatial spread and intermingling of several genets (see Figure 2). Distances of 80 m and more between ramets of the same clone can hardly be explained by first generation asexual establishment. Thus, CWS may provide the conditions for the formation of three local generations starting with a sexual generation followed by at least two asexual generations.

These aspects were not taken into account by [4] and [15] because almost young trees (10–30 years) were analysed. Further, these findings rely heavily on the applied methods of clonal analysis. As [4] emphasized, markers more variable than isozymes are required to describe wild cherry clone sizes. Our study, however, demonstrates that high variability alone may not be a sufficient condition for reliable estimates of clonal propagation. What is needed in addition is a well reasoned assumption on the amount of gene association. Based on our newly developed "Type one error method", it turned out in our study that even small deviations from the assumption of the absence of gene associations may imply sizable errors in the identification of clones. This goes along with differences for gene association among genotypes. The apparently small values of gene association inferred from our observations constitute lower limits of association. It can therefore not be ruled out that the actual degrees of gene association are distinctly larger.

Clonal propagation is also likely to affect sexual reproduction if it occurs at sizable proportions (in terms of C-values). In this case mating and thus seed set may be strongly reduced as a consequence of the gametophytic incompatibility system. It is interesting to note in this concern that the stand of Wibbecke with its distinctly larger C-value was observed to show only sporadic seed set since the last eight years.

The effect of clonal propagation on genetic variation is analysed with the help of measures of genetic diversity in both the gene and the hypothetical gametic pool. At a first site it may be surprising that the stand with the higher *C*-value (Wibbecke) also showed higher gene as well as hypothetical gametic diversity. Replacement of genetically variable individuals by copies of a single genotype can reasonably be expected to reduce overall haplotype variation. This should describe the situation in Wibbecke, since spontaneous repeated seedling recruitment is rarely observed within a stand of wild cherry. Because this species is extremely affected by browsing, quick recovering ability of root suckers from sublethal damage by herbivores may lead to mainly vegetative reproduction within local stands of *P. avium *[2]. Particularly within clumps of wild cherry occurrences, a genotype that consists of dozens of ramets is more likely to have some ramets escape herbivory than a genotype that consists of a single stem [16].

Accordingly, in a review of [1] surprise is expressed about the fact that populations of clonal plants can exhibit considerable levels of genetic variation, comparable with those found for populations of non-clonal plants [17-20]. Especially in stable habitats, any difference in cloning success of particular genets as well as random drift should lead to a decrease in genet number over time and dominance of a few clones, such that genetic variation decreases [1]. This situation can be assumed to be realized in CWS and could thus apply to our observations in *P. avium*.

The difference in size between the two stands and random events during establishment of the stands can be drawn upon for an explanation of the observations on genetic diversity. Indeed, there are fewer individuals in Roringen, and this corresponds to its smaller haplotype (gene and gamete pool) diversity. Yet, this contrasts with the fact that the number of genotypes in Roringen is twice the number of genotypes in Wibbecke. A closer look at the putative clones in Wibbecke reveals that the clone with the by far largest number of ramets has the second largest degree of heterozygosity. This explains the larger haplotype diversity in Wibbecke. In a more general context, the present observation suggests heterozygote advantage in clonal propagation as an interesting hypothesis to test in future studies, when asexual reproduction is associated with unusually large degrees of haplotype diversity (see also [21]).

## Conclusion

Even though the study had to be limited to one stand for each of the two forest management systems, our results provide useful guide lines for more comprehensive investigations.

1. Clear differences were found in the reproduction pattern of two *P. avium *(wild cherry) stands. Particularly in the CWS, individual cloning success via root suckering was observed to exist at distinctly higher degrees compared to our investigations in a present-day HFS (group selection shelterwood system) which is believed to represent (near-) natural forest reproduction patterns. Further, the local formation of more than one asexual generation may be characteristic for CWS as is suggested by the large spatial spread and intermingling of several genets. An interesting aspect for more detailed investigations is to be seen in the fact that a reduction in genet number in combination with the spatial distribution of genets may lower mating opportunities due to the species' GSI system. This is in accordance with our observations of a reduced seed set in the CWS. Since the latter aspect applies more generally to fertilization success of self-incompatible tree species, it can be expected to have a significant effect on the sustainability of different forest management systems. The results obtained justify further studies on these topics, particularly if rare and endangered tree species of the *Rosaceae *family with similar reproductive features are considered.

2. This study demonstrates the importance of considering gene associations in addition to genic variation at individual loci in studies of multilocus genotypic structures. The newly developed "Type one error" method allows quantifying the lower level of multilocus gene associations within populations. In fact, this method may turn out to provide new insights into reproductive characteristics of species that have so far not been considered reproducing asexually. The method is also open to optimization, concerning the estimation of multilocus gene associations with the perspective of allowing more comprehensive insights into the organization of larger parts of the genome.

3. The observation that the largest genet is the one with the second largest degree of heterozygosity gives rise to speculations about associations between cloning success and degree of heterozygosity for cloning success. The fact that we found ramets of the same clone or genet separated by comparatively large distances suggests that these ramets are subject to larger microenvironmental heterogeneity. This in turn has been frequently argued to promote heterozygosity. If this would turn out to be relevant, it would suggest that cloning ability has an effect on heterozygosity comparable or even stronger than the GSI mating system (for an estimation the effect of the mating system see [22]).

## Methods

### Plant material

Because of limited resources we had to make a choice between sampling several stands for both management systems at a low level of individual resolution, since experimental effort is particularly large for forest tree species with a very low density, or to concentrate on an exhaustive survey of one exemplar of each system. We had to decide in favour of the latter, since locally complete surveys are required for reliable estimates of degrees of clonal reproduction. For this reason, the present study is to be understood as a first attempt to test common hypotheses on the effects of different management types on the breeding system of wild cherry and to infer more realistic hypotheses from these observations.

Two *P. avium *stands near Göttingen in Germany were chosen, both of which are spatially isolated from other occurrences (because of larger surrounding agricultural areas). The two stands originate from natural regeneration but have been subjected to different silvicultural treatments. All wild cherry trees were sampled within these two stands (figure 2) in order to avoid effects of differences in sample size on the accuracy in allele frequency estimates and thus the method of clonal identification in each plot.

One of the chosen stands is a mixed stand near the village of Wibbecke that consists of 78 wild cherry trees that are about 100 years old. These cherry trees are scattered among a mixture of predominantly sessile oak (*Quercus petraea*) and hornbeam (*Carpinus betulus*) as well as sporadically occuring Norway maple (*Acer platanoides*), field maple (*Acer campestre*) and wild service tree (*Sorbus torminalis*). Altogether, the area of the stand covers more than 10 hectares. Referring to historical evidence, the trees had been managed as CWS until the beginning of the last century. This is also the reason for the almost total absence of beech (*Fagus sylvatica*). The forest management plan of 1991 mentioned that most of the wild cherry trees might be asexually regenerated by coppicing.

The second wild cherry stand (56 trees on about 5 hectares) near Roringen is about 70 years old and located in a protection area that is treated as a high-forest system. The consequence is that the area is mostly dominated by beech, although the environmental conditions are very similar to Wibbecke (chalcerous soil). Additional scattered tree species are common ash (*Fraxinus excelsior*), hornbeam (*C. betulus*), Norway maple (*A. platanoides*), field maple (*A. campestre*) and, very scattered, wild service tree (*S. torminalis*). The observed tree mixture is representative for the group of mesophyllic chalcerous beech forests.

### DNA extraction and visualization of amplified SSR (microsatellite) fragments

DNA was purified from fresh leaf material using the QIAGEN DNeasy96 Plant Kit and tested on a 0.8% agarose gel. For standard population genetic studies six nuclear microsatellites were analysed, as described in table 1. Amplification was carried out in a PTC-200 (MJ Research) using labeled primers with green (HEX) or blue (FAM) flourescent dyes. The Polymerase Chain Reaction (PCR) was performed in a 10 μl reaction volume containing 10 ng template DNA, 1.5 mM MgCl_2_, 10 mM Tris-HCl pH 9.0, 50 mM KCl, 0.15 mM of each dNTPs (QIAGEN), 0.5 units of HotStarTaq™ DNA polymerase (QIAGEN) and 0.2 μM of each primer. SSR fragments were visualized on the ABI PRISM 3100 Genetic Analyser (Applied Biosystems/HITACHI) and analysed using GeneScan 3.7 and the Genotyper 3.7 computer software. Since this investigation was no large-scale genotyping project, we manually checked all the automatically processed genotypes in order to exclude errors of PCR product size analyses (problems for high-throughput see [23]). The SSR allele sizes in our study could be determined uniquely.

### Measurement of genetic diversity

To give an idea about the range of genetic variation available for the analysis of clonal propagation, the *effective number of alleles *[24], the *gene pool diversity *[25] and the *hypothetical gametic diversity *[26] are communicated. These parameters give hints as to the efficiency of clone identification in the sense that higher diversity values are generally expected to detect existing clones with higher probability.

### Statistical and conceptual considerations

#### Testing for clonal reproduction

The testing procedure for clonal propagation is based on the idea that exclusively sexual reproduction of a genotype is unlikely, if its observed frequency exceeds a specified threshold. The (hypothetical) threshold frequency is argued to typically not be exceeded in a population with specified frequencies of the genes represented in the genotype, and in which this genotype is exclusively sexually produced. Hence, given the threshold frequency for the genotype, the hypothesis that all of the copies of the genotype observed in a sample result from sexual reproduction is rejected if the number of copies is too large. More precisely, the hypothesis is rejected if the probability *C*_*n*_^*N*^(*H*) of finding the observed number *n *or more copies of the genotype in a sample of size *N *and for given (hypothetical) threshold frequency *H *is smaller than a given significance level ε, i.e. if

CnN(H)=∑i=nN(Ni)Hi(1−H)N−i<ε
 MathType@MTEF@5@5@+=feaafiart1ev1aaatCvAUfKttLearuWrP9MDH5MBPbIqV92AaeXatLxBI9gBaebbnrfifHhDYfgasaacH8akY=wiFfYdH8Gipec8Eeeu0xXdbba9frFj0=OqFfea0dXdd9vqai=hGuQ8kuc9pgc9s8qqaq=dirpe0xb9q8qiLsFr0=vr0=vr0dc8meaabaqaciaacaGaaeqabaqabeGadaaakeaacqWGdbWqdaqhaaWcbaGaemOBa4gabaGaemOta4eaaOGaeiikaGIaemisaGKaeiykaKIaeyypa0ZaaabCaeaadaqadiqaauaabeqaceaaaeaacqWGobGtaeaacqWGPbqAaaaacaGLOaGaayzkaaaaleaacqWGPbqAcqGH9aqpcqWGUbGBaeaacqWGobGta0GaeyyeIuoakiabdIeainaaCaaaleqabaGaemyAaKgaaOWaaeWaceaacqaIXaqmcqGHsislcqWGibasaiaawIcacaGLPaaadaahaaWcbeqaaiabd6eaojabgkHiTiabdMgaPbaakiabgYda8iabew7aLbaa@4D17@

In essence, *C*_*n*_^*N*^(*H*) <*ε *rejects the hypothesis that the frequency of the genotype in question is equal to or smaller than the threshold frequency *H*. Rejection of the hypothesis thus implies that at least two copies of the genotype can be assumed to belong to the same clone. Hence, if additional gene loci are considered in the sample and if at these loci up to *n *- 1 different genotypes are found, this is still in accordance with the rejected hypothesis. By definition, the hypothesis is not rejected, and thus exclusively sexual reproduction of the genotype is assumed for all threshold frequencies *H *for which *C*_*n*_^*N*^(*H*) ≥ *ε*[10].

#### Testing genotype copies for representing a single clone

**Figure 3:**
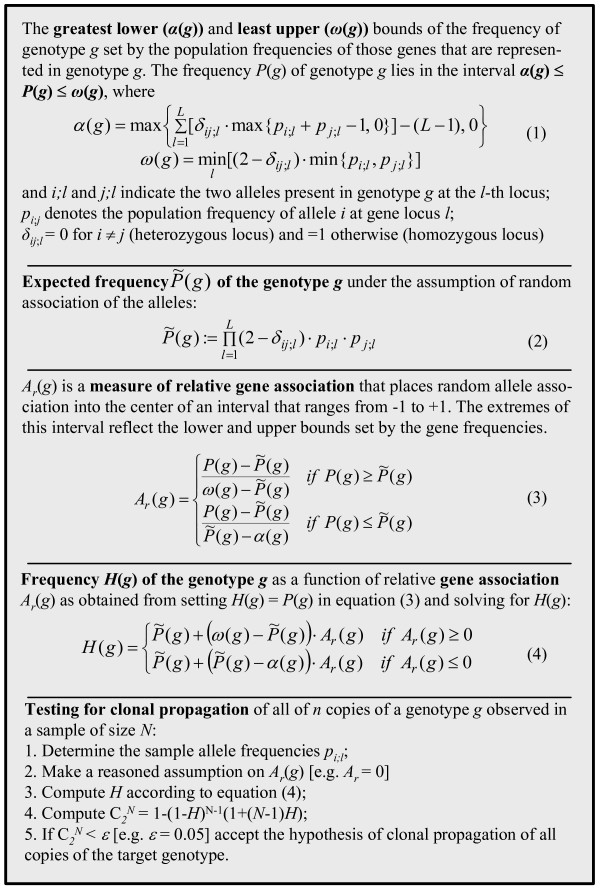
The statistical basis for testing asexual vs. sexual propagation considering gene associations (according to [10], changed).

In most cases, as in the present paper, the focus is set on knowing whether *all *of the copies of a genotype observed in a sample could belong to the same clone. This hypothesis is accepted if any two individuals which carry copies of the genotype are unlikely to belong to different clones (genets), i.e. if *C*_*2*_^*N*^(*H*) <*ε *(see bottom of figure 3, for the underlying theory see [10]). Hence, *C*_*2*_^*N*^(*H*) ≥ *ε *implies that the hypothesis cannot be rejected that two of the copies of the genotype belong to different clones including the case where all copies result from sexual reproduction. In the current literature, the almost exclusively considered case of sexual reproduction is specified by complete random association of genes such that the threshold frequency *H *of the target genotype results from multiplication of the frequencies of those alleles that are represented in the genotype [10]. Because of this fact our analysis will start with this assumption of the threshold frequency *H *and will later on check its appropriateness.

#### Utilizing Type one errors in the assessment of gene associations

As was explained in the introduction, the assumption of random gene association as a characteristic of sexual reproduction is generally difficult to justify. Thus, the rejection of sexual reproduction of a set of individuals with identical multilocus genotypes may be erroneous if the assumption on gene association is incorrect. Accepting clonal propagation of a genotype could in this case be an error. This is called a Type one error, and it erroneously rejects the hypothesis of sexual reproduction.

Sexual reproduction is characterized by a specific threshold frequency of a genotype under consideration, as explained above. Therefore, if a Type one error is committed, this is due to an inappropriate characterization of sexual reproduction as determined by the threshold frequency. A major problem in the assessment of gene associations at multiple loci results from limited sample sizes in relation to the very small frequencies expected for multilocus genotypes. Therefore we developed a non-conventional approach to assessing gene associations that does not directly depend on frequency estimates of genotypes:

The possibility of committing a Type one error under the hypothesis of random association of genes in genotypes is checked in the present paper by additionally considering two microsatellite loci, which were scored in the stand of Roringen in connection with a different study, and six polymorphic isozyme systems (*pgm*, *idh*, *6-pgdh*, *skdh*, *got *and *aco*) in the stand of Wibbecke, which were studied earlier by [27]. Detection of a Type one error with the help of these loci and on the basis of *C*_*2*_^*N*^(*H*) will then give rise to the reconsideration of random association of genes as an appropriate specification of the threshold frequency *H*. As was explained above, in this reconsideration *H *must be large enough to guarantee *C*_*2*_^*N*^(*H*) ≥ *ε*, and, in order to be admissible, it must respect the constraints set by the allele frequencies in the population. Taking account of these constraints, genotype frequencies can be expressed as a function of the frequencies of those alleles represented in the genotype and of a consistently definable measure *A*_*r*_(*g*) of relative gene association (see figure 3). *A*_*r*_(*g*) varies between -1 and +1 and becomes zero exactly for the case of random association of all genes representted in genotype *g*.

For given gene frequencies, the (hypothetical) frequency *H*(*g*) of a genotype increases strictly with the measure *A*_*r*_(*g*) of gene association, as does the significance probability *C*_*n*_^*N*^(*H*) for each fixed *n *and *N*. Hence, as *A*_*r *_moves from -1 to +1, the significance probability *C*_*2*_^*N *^may cross the significance level *ε*, so that for small *A*_*r *_exclusively clonal propagation is inferred and for sufficiently large *A*_*r *_sexual reproduction cannot be rejected in producing the observed copies of the genotype. Consequently, if the hypothesis of exclusively clonal propagation is accepted under a special assumption on *A*_*r *_(*A*_*r *_= 0 say, as is common usage) and if consideration of additional genetic traits reveals genetic variation within the supposed clone, then a Type one error is detected that is likely to be due to an inappropriate specification of *A*_*r*_. In conclusion, a higher degree *A*_*r *_of gene association must be assumed to avoid at least the detected Type one error, and this degree must be large enough to yield a threshold genotype frequency *H *for which *C*_*2*_^*N*^(*H*) ≥ *ε*. The thus obtained value of *A*_*r *_is likely (on a level 1 - *ε *of likelihood) to constitute a lower bound for the degree of gene association characteristic of the observed genotype.

The principle of this approach of assessing degrees of gene association can be extended by picking one of the studied gene loci and considering it as an "additional" locus in the above sense, while the remaining loci are used for the primary characterization of multilocus genotypes. If one of these genotypes is observed in *n *≥ 2 copies, which however differ at the "additional locus", the smallest value of *A*_*r *_can be determined such that for the corresponding hypothetical threshold frequency *H *one obtains *C*_*2*_^*N*^(*H*) ≥ *ε*. This value of *A*_*r *_specifies a lower bound for the degree of gene association of the target genotype on a level 1-*ε *of likelihood.

Mutation is, of course, another explanation of detecting genetic differences when considering additional gene loci. In this case it would be erroneous to conclude a Type one error, and the above method of estimating degrees of gene association would be without substance. In our study, however, we analysed tissue samples of several branches per adult tree without finding any genetic variation within trees. This leaves us with the possibility of mutation in primordial cells of the root. It would therefore require very large numbers of ramets of one clone in order to reach a sizable probability of detecting a mutational event. Such numbers of genetically identical individuals are not observed in our study. It is thus not reasonable to consider mutation as a significant force in our study.

### Determination of the degree of clonal propagation

The degree of clonal propagation is measured as the average number of individuals (ramets) per clone or genet, where non-cloned individuals count as one clone or genet (*N*/*G*, with *N *= number of individuals and *G *= number of clones or genets). The occasionally reciprocal *G*/*N *is used in place of *N*/*G *(see e.g. [17,28]). Because of its higher intuitive appeal we however prefer the average number of ramets per genet as a measure of the individual genet cloning success.

On the stand level we used Simpson's index *C *of concentration (see [29], p. 309) for measuring the degree to which a stand results from clonal reproduction. This parameter calculates the probability that two individuals (drawn randomly from a population of *N *individuals without replacement) are identical in their multilocus genotypes: *C *= {[*n*_*i *_(*n*_*i *_- 1)]/[*N *(*N *- 1)]}, where *n*_*i *_is the number of individuals of genotype *i *and *N *is the total number of individuals in the population. Thus, *C *= 1 if the whole stand consists of one genet (clone) and *C *= 0 in the absence of clonal propagation.

## Authors' contributions

This paper is the result of intense cooperation between both authors on all topics. AMH carried out field work as well as molecular genetic studies. AMH and HRG participated in the design of the study and in the performance and interpretation of the statistical analysis. Both authors read and improved the final manuscript.

## Figures and Tables

**Table 1:** Characteristics of the analysed microsatellite loci

				No. of alleles		No. of effective alleles	
Locus code	Repeat motif	Annealing temperature	Length of alleles (bp)	Ror	Wib	Ror	Wib
UDP96-005*	(AC) & (TC)	61°C	110–156	5	5	3.47	2.59
UDP98-021*	(TG) & (AG)	58°C	100–114	3	4	2.01	1.51
UDP98-410*	(GA)	52°C	116–138	6	5	2.56	3.85
UDP98-412*	(AG)	60°C	87–129	7	7	3.90	4.88
BPPCT 034**	(GA)	56°C	222–260	7	7	4.02	4.57
BPPCT 040**	(GA)	56°C	116–142	5	5	2.48	3.89
		gene pool diversity				2.88	3.00
		hypothetical gametic multilocus diversity				694.20	1 306.24

Microsatellite locus code according to [11]* and [12]**, the optimized annealing temperature, the range of size variation in basepairs (length of alleles), the number of alleles found at each locus, the effective number of alleles *v*_*2 *_according to [26] and [24, p. 324]; the gene pool diversity and the hypothetical gametic multilocus diversity *v*_*gam*_; Ror and Wib are the acronyms for the two analysed *P. avium *stands in Roringen and Wibbecke

**Table 2:** Allele frequencies detected at the six microsatellite loci in the two *P. avium *stands

	Allele frequencies			Allele frequencies	
Locus code/Alleles	Ror	Wib	Locus code/Alleles	Ror	Wib
UDP96-005			UDP98-021		
1	0.036	0.237	1	0.661	0.795
2	0.393	0.160	2	0.000	0.032
3	0.286	0.019	3	0.205	0.167
5	0.071	0.019	5	0.134	0.006
6	0.214	0.564			
					
UDP98-410			UDP98-412		
2	0.214	0.308	1	0.134	0.071
4	0.571	0.237	2	0.009	0.019
5	0.062	0.288	3	0.045	0.141
6	0.009	0.000	5	0.232	0.224
7	0.116	0.006	6	0.384	0.321
8	0.027	0.160	7	0.009	0.083
			8	0.187	0.141
					
BPPCT 034			BPPCT 040		
1	0.018	0.064	1	0.045	0.090
2	0.375	0.321	2	0.143	0.276
3	0.134	0.167	3	0.571	0.064
4	0.009	0.250	4	0.232	0.231
6	0.071	0.026	7	0.009	0.340
7	0.134	0.141			
8	0.259	0.032			

All individuals of Roringen (Ror) and Wibbecke (Wib) are included in the calculations, also individuals with identical multilocus genotype

**Table 3:** Testing of identical multilocus genotypes for clonal propagation based on the absence of gene associations

**Multilocus-Genotype (MLG)**	No. of individuals with identical MLG	*C*_*n*_^*N*^	*C*_*2*_^*N*^	detected type one error marker type (locus)
*Ror1*	3	1.3793· 10^-10^	2,2898· 10^-07^	*SSR (UDP96-001; UDP98-411)*
*Ror2*	3	2.2205· 10^-15^	2,4674· 10^-11^	
*Ror3*	2	3.8747· 10^-07^	3,8747· 10^-07^	
*Ror4*	4	5.0660· 10^-13^	1,8033· 10^-06^	
*Wib1*	8	3,8858· 10^-15^	3,8320· 10^-06^	*isozymes (pgm, idh, 6-pgdh, skdh, aco)*
*Wib2*	2	8,4748· 10^-11^	8,4748· 10^-11^	
*Wib3*	7	2.6645· 10^-15^	1,9343· 10^-07^	*isozymes (6-pgdh, pgm, skdh)*
*Wib4*	6	0	3,2969· 10^-07^	*isozyme (idh)*
*Wib5*	3	5.4934· 10^-13^	1,1209· 10^-08^	
*Wib6*	7	0	7,9074· 10^-06^	*isozymes (idh, skdh)*
*Wib7*	14	4.3299· 10^-15^	1,1990· 10^-08^	
*Wib8*	3	1.8541· 10^-14^	1,1904· 10^-09^	
*Wib9*	2	1.6718· 10^-11^	1,6718· 10^-11^	
*Wib10*	2	6,8013· 10^-08^	6,8013· 10^-08^	
*Wib11*	3	0	3,1075· 10^-13^	
*Wib12*	5	0	8,8324· 10^-12^	
*Wib13*	3	3.1086· 10^-15^	1,5363· 10^-10^	

Multilocus genotypes (MLG) represented by at least two individuals in the *P. avium *stands Roringen (Ror1 to Ror4) and Wibbecke (Wib1 to Wib13), the total number of individuals with the determined identical MLG, the statistical values *C*_*n*_^*N *^and *C*_*2*_^*N *^for testing clonal vs. sexual reproduction and the marker type that detected a Type one error

**Table 4:** Results of the Type one error analysis

***Multilocus-Genotype (MLG)***	P˜ MathType@MTEF@5@5@+=feaafiart1ev1aaatCvAUfKttLearuWrP9MDH5MBPbIqV92AaeXatLxBI9gBaebbnrfifHhDYfgasaacH8akY=wiFfYdH8Gipec8Eeeu0xXdbba9frFj0=OqFfea0dXdd9vqai=hGuQ8kuc9pgc9s8qqaq=dirpe0xb9q8qiLsFr0=vr0=vr0dc8meaabaqaciaacaGaaeqabaqabeGadaaakeaacuWGqbaugaacaaaa@2DE4@(*g*)	*ω *(*g*)	*A*_*r*_(*g*) for *C*_*2*_^*N *^= 0.05	*C*_*2*_^*N*^for *A*_*r*_(*g*) = 0,0275
*Ror1*	1.2196· 10^-5^	0.232	0.0275	/
*Ror2*	1.2658· 10^-7^	0.054	/	0.0032
*Ror3*	1.5866· 10^-5^	0.090	/	0.0087
*Ror4*	3.4244· 10^-5^	0.268	/	**0.0649**
*Wib1*	3.5754· 10^-5^	0.282	0.0162	/
*Wib2*	1.6788· 10^-7^	0.071	/	0.0104
*Wib3*	8.0274· 10^-6^	0.282	0.0162	/
*Wib4*	1.0481· 10^-5^	0.166	0.0275	/
*Wib5*	1.9321· 10^-6^	0.038	/	0.0031
*Wib6*	5.1381· 10^-5^	0.282	0.0161	/
*Wib7*	1.9983· 10^-6^	0.224	/	**0.0830**
*Wib8*	6.2936· 10^-7^	0.052	/	0.0057
*Wib9*	7.4621· 10^-7^	0.083	/	0.0139
*Wib10*	4.7596· 10^-6^	0.282	/	**0.1231**
*Wib11*	1.0203· 10^-8^	0.038	/	0.0031
*Wib12*	5.4225· 10^-8^	0.064	/	0.0085
*Wib13*	2.2618· 10^-7^	0.038	/	0.0031

(1) the expected frequency P˜
 MathType@MTEF@5@5@+=feaafiart1ev1aaatCvAUfKttLearuWrP9MDH5MBPbIqV92AaeXatLxBI9gBaebbnrfifHhDYfgasaacH8akY=wiFfYdH8Gipec8Eeeu0xXdbba9frFj0=OqFfea0dXdd9vqai=hGuQ8kuc9pgc9s8qqaq=dirpe0xb9q8qiLsFr0=vr0=vr0dc8meaabaqaciaacaGaaeqabaqabeGadaaakeaacuWGqbaugaacaaaa@2DE4@(g) of the multilocus genotypes (MLG) in Table 3 under the assumption of random association of the alleles; (2) the least upper bound *ω *(*g*) of the frequency of the MLG set by the population frequencies of the alleles that are represented in this MLG; (3) the value of *A*_*r*_(*g*) for which *C*_*2*_^*N *^= *ε *= 0.05 (/denotes the absence of a Type one error); (4) the value of *C*_*2*_^*N *^for the relative gene association *A*_*r*_(*g*) = 0.0275 that would have avoided all of the observed Type one errors (values in bold exceed significance level *ε *= 0.05)
